# Treatment of Brain Metastasis from Lung Cancer

**DOI:** 10.3390/cancers2042100

**Published:** 2010-12-15

**Authors:** Alexander Chi, Ritsuko Komaki

**Affiliations:** 1 Department of Radiation Oncology, University of Arizona, 1501 N Campbell Ave., Tucson, AZ 85724, USA; E-Mail: achiaz2010@gmail.com; 2 Department of Radiation Oncology, University of Texas MD Anderson Cancer Center, 1515 Holcombe Blvd, Houston, TX 77030, USA

**Keywords:** brain metastasis, WBRT, SRS, lung cancer, PCI, neurotoxicity

## Abstract

Brain metastases are not only the most common intracranial neoplasm in adults but also very prevalent in patients with lung cancer. Patients have been grouped into different classes based on the presence of prognostic factors such as control of the primary tumor, functional performance status, age, and number of brain metastases. Patients with good prognosis may benefit from more aggressive treatment because of the potential for prolonged survival for some of them. In this review, we will comprehensively discuss the therapeutic options for treating brain metastases, which arise mostly from a lung cancer primary. In particular, we will focus on the patient selection for combined modality treatment of brain metastases, such as surgical resection or stereotactic radiosurgery (SRS) combined with whole brain irradiation; the use of radiosensitizers; and the neurocognitive deficits after whole brain irradiation with or without SRS. The benefit of prophylactic cranial irradiation (PCI) and its potentially associated neuro-toxicity for both small cell lung cancer (SCLC) and non-small cell lung cancer (NSCLC) are also discussed, along with the combined treatment of intrathoracic primary disease and solitary brain metastasis. The roles of SRS to the surgical bed, fractionated stereotactic radiotherapy, WBRT with an integrated boost to the gross brain metastases, as well as combining WBRT with epidermal growth factor receptor (EGFR) inhibitors, are explored as well.

## 1. Introduction

Brain metastases are the most common intracranial neoplasm, occurring in 8–10% of cancer patients, and are a significant cause of cancer-related morbidity and mortality worldwide [1,2] The incidence of brain metastases is rising with an annual incidence of approximately 170,000 to 200,000 in the United States [3]. This is caused by a combination of factors including the improved therapeutic efficacy of current cancer treatments, which leads to longer survival, such as the addition of bevacizumab to chemotherapy as the first-line treatment of metastatic non-small cell lung cancer; and failure in a potential systemic therapy sanctuary site, or more frequent brain surveillance for specific cancers that have a predilection for brain metastases; and improvements in modern imaging technology, which lead to the diagnosis of brain metastases at an earlier stage [4,5]. However, such an increase in the incidence of brain metastases in recent years has not been observed in all studies, and may possibly be attributed to under-diagnosis in earlier years [1,6]. The most common origins of brain metastasis include primary cancers of the lung, breast, skin (melanoma), and the GI tract. Among these, primary tumors in the lung are the most common cause of brain metastases, as up to 65% of patients with lung cancer will ultimately develop brain metastases [7].

As the leading cause of cancer mortality, and the most prevalent cancer in men, lung cancer accounted for an estimated 161,840 deaths in the United States with an incidence of 215,020 in 2008. Furthermore, approximately 1.35 million cases were diagnosed worldwide with 1.18 million deaths in 2002 [8]. Therefore, brain metastasis is a very important problem in the overall management of lung cancer. Among the various histologies, small cell lung cancer (SCLC) is the most likely to metastasize to the brain with an 80% probability of brain metastasis after two years from diagnosis [6]. Brain metastases develop in approximately 30% of patients with non-small cell lung cancer (NSCLC) [9]. Among the various histologies of NSCLC, the relative frequency of brain metastases in patients with adenocarcinoma and large cell carcinoma was much higher than that in patients with squamous cell carcinoma [10,11].

Most patients present with significant neurological signs and symptoms that are related to the location and extent of brain involvement. They include both focal neurological changes and general symptoms secondary to increased intracranial pressure [12]. Major clinical presentations are listed in Table 1 [13]. Contrast-enhanced MRI is the preferred imaging study for the diagnosis of brain metastases over non-enhanced MRI or computed tomography (CT) scans in detecting cerebral metastases and in differentiating metastases from other central nervous system (CNS) lesions [14,15]. The recommended pregadolinium studies include T2-weighted and T1-weighted sequences, and the recommended postgadolinium studies include the T1-weighted and fluid-attenuated inversion-recovery (FLAIR) sequences [5]. Thinner axial slices without skips may be necessary to detect the smallest lesions. If the diagnosis is still in doubt, biopsy should be considered. Brain metastases are usually found at the junction of the grey and white matters, with circumscribed margins and large amounts of vasogenic edema relative to the size of the lesion. Furthermore, they usually present as multiple lesions as a result of a lung primary [16].

Without treatment, the median survival of patients is 4–7 weeks [17,18,19]. The treatment can usually be divided into symptomatic and therapeutic strategies. Symptomatic relief is most commonly achieved with corticosteroids to reduce peritumoral edema and anticonvulsants to prevent recurrent seizures. Systemic steroids alone improve neurological function and prolong survival to approximately two months [20]. Whole brain radiotherapy (WBRT), as the primary treatment approach for brain metastases, improves neurological function and prolongs median survival to three to five months [12]. Due to the poor survival outcomes associated with brain metastases, more aggressive treatments for patients have been sought and investigated. In general, the therapeutic approach largely depends on the number and location of metastases, as well as the extent of extra-cranial tumor involvement. In the following sections, prognostic factors that may influence treatment selection and the various treatment approaches will be reviewed.

**Table 1:** Clinical presentation of brain metastasis.

Symptom	Percentage (%)	Sign	Percentage (%)
Headache	49	Hemiparesis	59
Mental problems	32	Cognitive deficits	58
Focal weakness	30	Sensory deficits	21
Ataxia	21	Papilledema	20
Seizures	18	Ataxia	19
Speech problems	12	Apraxia	18

## 2. Prognostic Factors

A retrospective recursive partitioning analysis (RPA) was performed based on three consecutive Radiation Therapy Oncology Group (RTOG) trials, which included approximately 1200 patients with brain metastases [21]. Three prognostic classes (RPA class I, II and III) were found to be associated with the overall survival of patients with brain metastases. This classification scheme is based on age at diagnosis, presence of extracranial disease, Karnofsky performance status (KPS), and the status of the primary cancer. RPA class I includes patients who are younger than 65 years of age, have a KPS score of ≥70, tumor controlled at the primary site, and no extracranial disease. RPA class III patients have a KPS score of less than 70. All other patients are in RPA class II. The median survival times for the RPA classes I-III were 7.1, 4.2, and 2.3 months, respectively. This RPA classification is the most commonly used prognostic system for brain metastases, with further validation in Phase III and major institutional studies for both NSCLC and SCLC [22,23,24,25]. Despite the common adaptation of RPA classification, clinicians are still faced with the dilemma of tailoring treatments to individual patients because factors such as the number or volume of brain metastases were not included in the RPA initially, estimation of systemic disease was not consistently reliable, *etc*. As newer data came out, a new prognostic index, the graded prognostic assessment (GPA), was generated based on data from five randomized RTOG studies involving brain metastases [26]. Please refer to Table 2 for details of the GPA scoring system. The median survival times according to GPA score were: GPA 0–1, 2.6 months; GPA 1.5–2.5, 3.8 months; GPA 3, 6.9 months; and GPA 3.5–4.0, 11.0 months (*p* < 0.05). The GPA prognostic index was further validated based on specific diagnosis at the primary site due to the heterogeneous response of brain metastases to various treatment approaches based on histology and the various patterns of systemic disease and response to systemic therapy for different types of primary tumor [27]. For both NSCLC and SCLC, all four prognostic factors remained significant, confirming the prognostic value of the original GPA for lung cancer.

**Table 2:** The graded prognostic assessment (GPA).

Score	0	0.5	1.0
Age	>60	50–59	<50
KPS	<70	70–80	90–100
No. of CNS metastases	>3	2–3	1
Extracranial metastases	Present	-	None

Abbreviations: KPS: Karnofsky performance score; CNS: central nervous system; No.: number

## 3. Symptomatic Management

The management of symptoms from brain metastases has primarily consisted of the usage of corticosteroids (e.g., dexamethasone or methylprednisolone) and anticonvulsants. Corticosteroids are given upon initial diagnosis to relieve the symptoms associated with peritumoral edema in approximately two-thirds of patients because of their ability to reduce the permeability of tumor capillaries [28,29]. In a study by Vecht *et al.*, doses of 8 *versus* 16 mg/day with tapering schedules over four weeks and doses of 4 *versus* 16 mg/day with continuation of these doses for 28 days prior to tapering demonstrated similar KPS improvements at seven days (54% to 70%) and 28 days (50% to 81%) in patients treated with WBRT and concurrent ranitidine [30]. However, patients in the 4 mg/day arm experienced a higher rate of drug reinstitution than in patients treated with 8 or 16 mg/day. Furthermore, the greatest KPS improvement was observed in patients in the 16 mg/day arm when this dose was tapered over four weeks. These findings suggest that greater KPS improvement arose from the maximal anti-inflammatory effects of the initial higher doses, while the late toxicity associated with corticosteroids was minimized with gradual tapering. A commonly used dexamethasone regimen in patients with brain metastases is a 10-mg intravenous (IV) bolus, followed by 4 to 6 mg PO every six to eight hours before gradual tapering with caution. However, initial corticosteroid use may be reserved for symptomatic patients owing to the common side effects of dexamethasone, including hyperglycemia, peripheral edema, psychiatric disorder, oropharyngeal candidiasis, Cushing’s syndrome, muscular weakness, and pulmonary embolism [31].

Approximately 15% of patients with brain metastases present with seizures, and seizure is frequently associated with supratentorial lesions. Seizures can be managed with antiseizure medications, but anticonvulsants are generally not given prophylactically. In a prospectively randomized study by Forsyth *et al.* [32], one hundred patients with newly diagnosed brain tumors were randomized to prophylactic anticonvulsants or no anticonvulsants. After a median follow up of 5.44 months, no difference in the rates of seizures at three months or seizure-free survival were observed, suggesting that antiseizure prophylaxis in brain tumor patients is not necessary.

## 4. Therapeutic Approaches

### 4.1. Whole Brain Radiotherapy

The palliative effects of WBRT for brain metastases were appreciated over half a century ago, and are widely accepted to extend the median survival of patients to three to six months, compared to one to two months without treatment [5]. Thus, WBRT continues to be the standard of care for patients with brain metastases, especially metastases from lung cancer. Multiple randomized studies have been conducted since the early 1970s to determine the optimal dose and fractionation of WBRT. Selected studies are summarized below in Table 3.

**Table 3:** Selected randomized trials of various dose fractionation regimens for WBRT.

	Dose/fractionation	*n*	Median Survival	*p* value
Borgelt/RTOG [33,34]				
First study (1971–1973)	30 Gy/10	233	21 wk	NS
	30 Gy/15	217	18 wk	
	40 Gy/15	233	18 wk	
	40 Gy/20	227	16 wk	
	10 Gy/1^*^	26	15 wk	
Second study (1973–1976)				
	20 Gy/5	447	15 wk	NS
	30 Gy/10	228	15 wk	
	40 Gy/15	227	18 wk	
	12 Gy/2^*^	33	13 wk	
Murray/RTOG 91-04 [35]	30 Gy/10	213	4.5 mo	NS
	54.4 Gy/34 (bid)	216	4.5 mo	
Haie-Meder/French [36]	36 Gy/6 split course^†^	106	5.3 mo	NS
	25 Gy/10	110	4.2 mo	
Priestman/Royal College of Radiology [37]	30 Gy/10	263	84 days	0.04
	12 Gy/2	270	77 days	

^*^ optional randomization in the 1st and 2nd RTOG study. ^†^ 18 Gy/3 split course with another 18 Gy/3 within one month. Abbreviations: WBRT: whole brain radiotherapy; wk: weeks; mo: months; NS: not significant

Over 50% of metastases in these studies were of lung origin. Various dose fractionation schedules were studied with no difference in any clinical outcome (*i.e*., survival times, symptomatic response rates, duration of symptomatic response). However, the ultrarapid schedules of 10 Gy in one fraction and 12 Gy in two fractions were shown to be associated with shorter remission periods, less time to progression of neurologic symptoms, and lower rate of complete disappearance of neurologic symptoms in the RTOG trials [34]. This suggests better palliative effects from the more prolonged schedules. Although a slight survival advantage may be seen with the 30 Gy/10 fractions regimen over the 12 Gy/2 fractions regimen, this is confined to patients with a good initial response [37]. Therefore, the dose fractionation schedule should be chosen based on patients’ prognosis, and the more prolonged dose fractionation schedules should be used for patients who are expected to live long enough to experience neurologic progression as well as the late radiation toxicity associated with large fraction sizes [38].

In the assessment of tumor response, a thorough imaging study of dose response based on tumor size and histology in 108 patients with 336 measurable lesions after WBRT (30 Gy in 10 fractions) was performed by Nieder *et al.* [39]. An overall response rate of 59% was observed at up to three months. Complete response rate by tumor type was 37% for SCLC, 25% for squamous cell carcinoma, and 14% for non-breast adenocarcinoma. An improved response rate was observed for smaller tumors without necrosis. In a separate study, the biologically effective dose (BED) was generated to compare different dose fractionation schedules by Nieder *et al.* [40]. Increasing BED was found to correlate with increased partial remission based on tumor size.

### 4.2. Surgery

In clinical practice, surgical resection is indicated for histological confirmation of diagnosis when the diagnosis is in doubt, and for immediate relief of neurological symptoms due to increased intracranial pressure [12]. Resection of a single brain metastasis has become a standard treatment option after the publication of several prospective studies evaluating the role of surgery combined with WBRT in the treatment of brain metastases [41,42]. In a prospective study of 48 patients by Patchell *et al.*, patients were randomly assigned to surgical removal of the brain tumor followed by radiotherapy or needle biopsy and radiotherapy [41]. Patients began WBRT 36 Gy/12 fractions within 14 days after surgery, whereas patients in the WBRT alone arm began radiotherapy within 48 hours of biopsy or study entry. The recurrence rates at the site of original metastasis for the surgery arm and the WBRT alone arm were 20% and 52%, respectively. The length of time from treatment to the recurrence of the original brain metastasis was significantly shorter for the WBRT alone arm than the surgical arm (median 21 *versus* >59 weeks, *p* < 0.0001). The median survival after surgery and adjuvant WBRT was much longer at 40 weeks *versus* 15 weeks with WBRT alone (*p* < 0.01). In addition, the patients in the surgical group maintained functional independence (KPS score of ≥70) much longer than the patients treated with radiation alone (median, 38 weeks *versus* 8 weeks, *p* < 0.005). The results from this study were confirmed in another study by Noordijk *et al.* [42], which demonstrated a median survival advantage with the addition of surgery (10 *versus* 6 months, *p* = 0.04). This survival advantage was most pronounced in patients with stable extracranial disease and patients ≤60 years old. In contrast, a study of 84 patients by Mintz *et al.* failed to demonstrate any survival advantage with surgery plus radiation [43]. This is most likely due to the fact that a significant proportion of the patients enrolled presented with active systemic disease and lower functional performance scores compared with the other two studies. The results from all three studies suggest that patients with a single brain metastasis and positive prognostic features, such as the control of extracranial disease and young age, will benefit more from surgical resection followed by WBRT compared with WBRT alone.

Surgery is usually limited to the dominant, symptomatic lesion in patients with multiple metastases. Surgery combined with adjuvant WBRT or stereotactic radiosurgery (SRS) have demonstrated similar survival outcome in patients with multiple lesions compared with patients with single brain metastasis in several single-institution studies [44,45,46]. Furthermore, the survival outcome from resection of all lesions approaches that from resection of a single lesion as shown by Bindal *et al.* [46]. Modern 30-day surgical mortality rates after resection of a single metastasis range from 0% to 10%. Surgical morbidity includes neurologic deficits (0% to 13%) and non-neurologic complications (0% to 20%) such as thromboembolism, and wound infections [5].

In a separate study, the benefit of adding WBRT after complete surgical resection of a single lesion (based on MRI at 2–5 days after surgery) was investigated by Patchell *et al.* [47]. The overall median follow up was 43 weeks in the observation group and 48 weeks in the radiation group. Postoperative WBRT was found to have superior local control (90% *versus* 54%; *p* < 0.001), distant intracranial control (86% *versus* 63%; *p* < 0.01), and overall intracranial control (82% *versus* 30%; *p* < 0.001) rates when compared with those who underwent surgical resection alone. However, no overall survival benefit was seen, despite the fact that patients who underwent WBRT were less likely to die from neurological causes than patients in the observation group (14% *versus* 44%; *p* = 0.003). The results of this study have recently been confirmed by a randomized Phase III study in Europe, EORTC 22952-26001 [48]. In this study, 359 patients were enrolled with non-progressing primary tumors that had metastasized to the brain. For all patients, brain metastases were initially treated with surgery or radiosurgery. Subsequently, the patients were randomized to prophylactic WBRT or observation. The median survival was 9.5 *versus* 10 months, respectively. Overall survival was 10.7 *versus* 10.9 months, respectively. However, WBRT was associated with superior progression-free survival (PFS), intracranial control, and fewer neurologic deaths. This may suggest an overall improvement in patients’ quality of life when WBRT is added to surgical resection.

### 4.3. Stereotactic Radiosurgery (SRS) with and without WBRT

SRS is a noninvasive technique that delivers a high dose of radiation to a precisely defined target volume through multiple coplanar or non-coplanar intersecting beams, as well as rotational arcs. This approach allows the center of the target to receive a very high dose relative to the surrounding normal brain tissue, as the intersecting beams achieve a very sharp dose gradient (dose fall-off). In recent years, SRS has emerged as an effective alternative to surgery for up to four small brain metastases. This is mainly due to the pseudospherical shape, well-defined margin, and the relatively small size of brain metastases at presentation [49]. As the lesions increase in size, the dose fall-off becomes less rapid, thus increasing the dose to the volume of normal brain immediately adjacent to the tumor. This increases the risk of edema and radiation necrosis, which may require surgery six months or longer after SRS. As a result, SRS is typically delivered to small lesions up to 4 cm in size [50]. The maximum tolerated dose (MTD) was determined in an RTOG Phase I dose escalation trial, RTOG 90-05, based on tumor size [51]. In this study, the MTD was found to be 24 Gy for lesions ≤2 cm, 18 Gy for lesions 2.1–3 cm, and 15 Gy for lesions 3.1–4 cm in maximum diameter. Each dose level was associated with incidence rates of grade 3–5 CNS toxicity of 14%, 20%, and 8%, respectively. The biological effects of SRS on tumors are largely unknown. However, recent studies have suggested the involvement of endothelial cell apoptosis, microvascular dysfunction, or the induction of a T-cell response against the tumor in addition to the radiation-induced DNA damage [52,53,54].

**Table 4:** Stereotactic radiosurgery (SRS) alone for brain metastasis from lung cancer.

	*n*	Median FU	Local Control	Median Survival
Zabel *et al.* [55]	80	6 mo	96% at 3 mo	4.5 mo
Kim *et al.* [56]	77(71 +WBRT)	8 mo	85%	10 mo
Williams *et al.* [57]	14/ 30	n/a	100%	7.9 mo
Sheehan *et al.* [58]	273	n/a	86%	Adeno: 10 mo
				Non-adeno: 7 mo
Sheehan *et al.* [59]	27	n/a	86%	4.5 mo
Mariya *et al.* [60]	84	8.5 mo	77% at 1 year	9 mo

Abbreviations: FU: follow up; WBRT: whole brain radiotherapy; mo: months

Among various studies, patients with good functional performance status, no active systemic disease, and longer time from the diagnosis of primary lung cancer often had better prognosis and lived longer (Table 4). Because of the excellent local control rates achieved by SRS, whether its addition to WBRT will lead to a survival benefit over WBRT alone has been investigated in many studies. This approach could be especially beneficial for patients who are not candidates for a craniotomy because of tumor location or existing medical contraindications. Three randomized studies have evaluated the efficacy of WBRT alone *versus* WBRT + SRS. Most patients had lung tumor histology in two of the published studies [61,62]. In the first randomized study by Kondziolka *et al.* [61], the local control rate at one year was found to be significantly better when SRS was added to WBRT in a small number of patients (92% *versus* 0%, *p* = 0.0016). However, no survival benefit was found with the addition of SRS. This study defined local recurrence as any increase in lesion size on MRI rather than the more usually employed RECIST (revised response evaluation criteria in solid tumors) system. In addition, this study was not controlled for corticosteroid use, radiation changes, or other factors possibly affecting the lesion size on MRI. Therefore, this study is difficult to interpret. The largest study done to date is the randomized controlled Phase III trial (RTOG-9508) of WBRT alone *versus* WBRT and SRS (Table 5). This RTOG trial enrolled 333 patients with one to three brain metastases and a KPS of ≥70 [62]. The primary end point was overall survival. No statistically significant difference in overall survival was found between the WBRT and SRS and the WBRT alone arms (6.5 and 5.7 months, respectively, *p* = 0.1356). However, WBRT and SRS led to a significant decrease in local recurrence at one year despite the fact that 19% of the patients initially assigned to the SRS boost arm did not receive SRS for various reasons (18 *versus* 29%, *p* = 0.01). In planned subgroup analysis, increased median survival (6.5 *versus* 4.9 months; *p* = 0.039) was associated with WBRT and SRS in patients with a single brain metastasis. An SRS boost also resulted in improvement in KPS and decreased steroid use at six months, suggesting an improvement in the quality of life with the addition of the SRS boost. This is a very important observation, as the primary objective of treatment in patients with brain metastases is the improvement of their quality of life since their overall survival is often very poor. In an unplanned subgroup analysis, an OS benefit was associated with RPA class I, tumor size ≥2 cm, and squamous/NSCLC histology. However, these three cohorts are exploratory subsets that required an adjusted *p* value of 0.0056 to reach significance [63]. On multivariate analysis using Cox regression, however, RPA class I for both single and multiple lesions and lung primary histology for multiple lesions were found to be significant beneficial prognostic factors. Overall, this study is considered by most to be a negative trial with regard to major end points for multiple metastases. The third study is a three-arm study (SRS, SRS and WBRT, and WBRT alone) from Brown University, reported in abstract form [64]. Superior local control and fewer brain metastases were reported with the addition of an SRS boost. However, no *p* value was reported, nor was any attempt made to stratify for further surgery, which would have made this a six-arm trial (the size of this trial would not be large enough to support a meaningful analysis for this reason). Furthermore, the SRS dose was unconventional because the tumor dose was not individualized based on tumor size or volume. All of these methodological flaws made this study difficult to interpret.

**Table 5:** Randomized study of SRS boost for patients with brain metastases, Radiation Therapy Oncology Group (RTOG) 95-08.

	WBRT + SRS	WBRT alone	SRS alone	*p* value
(n = 333; 1–3 lesions)				
Primary end point: Overall survival		37.5 Gy/15 fx		
1–3 lesions	5.7 mo	6.5 mo		NS
Single brain metastasis	6.5 mo	4.9 mo		0.04
(planned subgroup analysis)				
Secondary end points				
Local control (1 year)	82%	71%		0.01
Neurologic death rate	28%	31%		NS
Performance outcome				
KPS stable/improved				
at 3 months	50%	33%		0.02
at 6 months	43%	27%		0.03
Mental status				NS
Unplanned subgroup analysis: Overall survival				
Largest tumor > 2 cm	6.5 mo	5.3 mo		0.04
RPA class I	11.6 mo	9.6 mo		0.05
Squamous/NSCLC	5.9 mo	3.9 mo		0.05
Other Outcomes				
Response rate (3 mo)				
Tumor	73%	62%		0.04
Edema	70%	47%		0.002

Abbreviations: SRS: stereotactic radiosurgery; WBRT: whole-brain radiotherapy; fx: fraction; NS: not significant; KPS, Karnofsky performance score; RPA: recursive partitioning analysis; NSCLC: non-small cell lung cancer; mo: months

The role of adjuvant WBRT after SRS was investigated in a randomized Phase III trial, Japanese Radiation Oncology Study Group 99-1, by Aoyoma *et al.* [65]. The primary study end point was overall survival, but the study was not powered to detect any overall survival difference. This study randomized 132 patients with one to four brain metastases to SRS alone or SRS and WBRT. No survival difference was detected (8.0 *versus* 7.5 mo for SRS *versus* SRS and WBRT, *p* = 0.42). The one year intracranial failure rate was decreased with the addition of WBRT (46.8% *versus* 76.4%, *p* < 0.001). More importantly, the average time to deterioration based on the Mini-Mental Status Examination (MMSE) was 16.5 months in the SRS + WBRT arm and 7.6 months in the SRS alone arm (*p* = 0.05) [66]. The results from this study suggest that WBRT can decrease brain failure and its neurological sequalae when added to SRS.

To date, only limited investigations have directly compared surgery and SRS for asymptomatic patients with good functional performance status and limited numbers of brain metastases. In the randomized Phase III study by Roos *et al.*, SRS and surgery were compared in the setting of adjuvant WBRT [67]. However, this study was closed owing to the slow accrual of only 19 patients. Due to the limited number of patients, no difference in CNS failure-free survival, overall survival, or intracranial control was found. In another randomized Phase III study by Muacevic *et al.*, 70 patients were randomized to SRS or microsurgical resection plus WBRT [68]. The inclusion criteria were: single brain metastasis of ≤ 3 cm in an operable site, KPS ≥ 70, and controlled extracranial disease with a life expectancy of at least four months. This study was also closed prematurely due to poor accrual. The final analysis of 64 patients demonstrated no difference in terms of survival, neurological death rates, and local control. However, patients in the SRS alone group did experience more distant recurrences (*p* = 0.04). This difference was lost after the effects of salvage SRS were accounted for. SRS was associated with shorter hospital stays, less frequent and shorter timed steroid application, and less acute low grade toxicity. But no difference in toxicity profile and quality of life were observed six months after treatment due to the small numbers of patients experiencing adverse effects at that time (SRS: 2, Surgery + WBRT: 6). Due to lack of adequate accrual, no firm conclusions can be made with regard to selection criteria for SRS ± WBRT or surgery and adjuvant WBRT in patients with operable single brain metastasis. We believe that the selection of SRS or surgical resection as initial therapy depends on the size, location, and the presentation of neurological symptoms, as well as each institution’s own policies. These two approaches are complementary in nature and are feasible for most patients as alternative treatment options.

### 4.4. Systemic Therapy and Radiosensitization

Other approaches to enhance the management of brain metastases have been investigated owing to the poor outcome after WBRT. As shown by Patchell *et al.*, the intracranial recurrence rate after a median follow up of 15 weeks in patients with single brain metastasis treated with WBRT alone was 52% [41]. This rate could be worse in the setting of multiple brain metastases. Such investigations are especially important in the treatment of lung cancer, as it has the highest incidence of brain metastases among all malignancies. In fact, a primary lung cancer can be assumed in 30–70% of patients who have a single brain metastasis [69]. The systemic treatment of brain metastases has generally been difficult owing to the effectiveness of the blood-brain barrier in preventing most chemotherapeutic agents from reaching the CNS. However, the blood-brain barrier may be disrupted when the tumor grows to a certain size, leading to neo-angiogenesis of more permeable vessels. These changes can usually be seen on CT or MRI as the accumulation of contrast medium and the development of edema. In fact, response rates of brain metastases to chemotherapy alone of 43% to 100% for metastases from SCLC and 0% to 38% for metastases from NSLCL have been observed in small single-institution Phase II studies [69]. This has led to a series of prospective studies investigating the radiosensitizing effects of various systemic agents (Table 6). However, no such agent has demonstrated any survival benefit thus far, but several agents have demonstrated increased response rates when combined with WBRT: temozolomide (an oral alkylating agent), nitrosourea + tegafur (masked compound of 5-fluorouracil), motexafin gadolinium (a metallotexaphrin that localizes within tumors more than in normal tissues), and efaproxiral (an allosteric modifier of hemoglobin that leads to increased oxygen release into tissue). Overall, there is no strong evidence supporting the use of radiosensitizers with WBRT in current clinical practice.

**Table 6:** Selected randomized studies on radiosensitizers for multiple brain metastases.

First Author/Study Group	Arms	Response Rate	*p* Value	Median Survival (months)	*p* Value
Komarnicky/RTOG 79-16^•^ [70]	30 Gy/10 fx	45%	NS	4.5	NS
	30 Gy/6 fx	42%		4.1	
	30 Gy/6 fx + misonidazole	45%		3.1	
	30 Gy/10 fx + misonidazole	45%		3.9	
Phillips/RTOG 89-05 [71]	37.5 Gy/15 fx	12%^*^	NS	6.12	NS
	37.5 Gy/15 fx + BrdUrd	27%^*^		4.3	
Ushio/Japan [72]	40 Gy (1.5-2 Gy/fx)	36%	< 0.05^†^	27	NS
	40 Gy + CCNU	69%		29	
	40 Gy + CCNU + Tegafur	74%		30.5	
Robinet/France (NSCLC only) [73]	30 Gy/10 fx after cisplatin & vinorelbine	21%	NS	6	NS
	30 Gy/10 fx concurrent with cisplatin & vinorelbine	20%		5.25	
Neuhaus/Germany (NSCLC or SCLC) [74]	40 Gy/20 fx	14/47	NS	N/A	NS
	40 Gy/20 fx + topotecan	11/49			
Guerrieri/Australia (NSCLC only) [75]	20 Gy/5 fx	10%	NS	4.4	NS
	20 Gy/5 fx + carboplatin	29%		3.7	
Knisely/RTOG 01-18 [76]	37.5 Gy/15 fx	N/A		3.9	NS
	37.5 Gy/15 fx + thalidomide			3.9	
Mehta/International^‡^ [77]	30 Gy/10 fx	8.8 mo	0.004	5.8	NS
	30 Gy/10 fx + MGd	24.2 mo		5.1	
Suh/REACH study [78]	30 Gy/10 fx	41%	0.01^$^	4.4	NS
	30 Gy/10 fx + efaproxiral	54%		5.4	
Verger/Spain [79]	30 Gy/10 fx	54%	0.03^Ş^	3.1	NS
	30 Gy/10 fx + temozolomide	72%		4.5	
Antonadou/Greece [80]	40 Gy/20 fx	67%	0.017	7.0	NS
	40 Gy/20 fx + temozolomide	96%		8.6	

• % survival time in KPS 90-100 range; *best response; ^†^ RT *vs.* RT + CCNU + Tegafur only; ^‡^ response as neurologic progression in patients from North America; ^$^ response rates as shown are in lung/breast cancer patients only; ^Ş^ 90-day freedom from brain metastases;Abbreviations*:* RTOG: Radiation Therapy Oncology Group; fx: fractions; BrdUrd: bromodeoxyuridine; CCNU: lomustine; MGd: motexafin gadolinium; NS: not significant.

## 5. Neurocognitive Functioning after Brain Irradiation

WBRT is associated with many acute, subacute, and late side effects. The acute toxicities such as fatigue, hair loss, and skin reaction are mild and self-limiting. The late toxicities are usually observed in patients with limited brain metastases and well controlled extracranial disease because these patients tend to survive longer. Late toxicities include diffuse white matter injury or cerebral atrophy and neurocognitive deficits. Neurocognitive function after cranial irradiation is being evaluated more closely as the efficacy of systemic therapy improves over time. This is of special importance in advanced stage lung cancer owing to the high frequency and short onset of brain metastases from lung cancer.

Neurocognitive impairment has been found frequently in long-term survivors of SCLC after prophylactic WBRT [81,82] and has been seen in patients with existing brain metastases as well. In a cohort of 98 patients with single brain metastasis, four of 38 patients (11%) who survived ≥ 1 year after postoperative WBRT developed severe dementia associated with ataxia and urinary incontinence [83]. All four patients were among a group of 23 patients (17%) who were treated with hypofractionated WBRT with fractions larger than 3 Gy/day. These toxicities were not observed in the patients who were treated with fractions ≤3 Gy/day. However, similar toxicities were seen in one patient who was treated with 3 Gy/day combined with intra-arterial chemotherapy. These findings suggest that large fractions and radiosensitizers, such as chemotherapy, may contribute to severe neurocognitive deficits in long-term survivors from brain metastases. However, such effects may not surface if the patients survive for less than one year. On the other hand, neurocognitive impairment was observed shortly after starting WBRT when patients underwent serial neurocognitive testing as shown by Welzel *et al.* [84]. But those authors recommended not avoiding WBRT since the neurocognitive dysfunction was restricted mainly to verbal memory. In addition, the risk of disease progression will always outweigh the risk of neurocognitive deficits secondary to brain irradiation since most recurrences can be associated with a neurologic deficit [85].

Some investigators believe that the neurocognitive outcome is directly related to intracranial tumor response after cranial irradiation, as neurocognitive deficits can be partially explained by intracranial tumor progression [86]. Furthermore, improvement in neurocognitive function in responding patients with multiple brain metastases also depends on the initial and posttreatment tumor volume [87,88]. In a study by Li *et al.*, a battery of standardized neurocognitive tests was administered monthly for six months and then every three months until death by trained and certified nurses or clinical research associates to patients with unresectable brain metastases who were treated with WBRT [88]. At two months, patients with greater tumor shrinkage were found to have longer median survival, higher survival rate at one year, and longer time to neurocognitive deterioration. The cognitive gain was especially prominent in executive function and fine motor coordination. Nine patients were alive at 15 months, and the correlation between tumor shrinkage and executive function as well as fine motor coordination persisted. Furthermore, neurocogntive function was found to be influenced mostly by disease progression early on after WBRT. The patients who became long-term survivors also experienced larger tumor volume reductions after WBRT, and they had the best neurocognitive outcome.

The combination of SRS and WBRT over WBRT alone has been supported by the results of RTOG 9508 for patients with single brain metastasis, good functional performance status, and no active extracranial disease [62]. No difference in neurological deaths or mental status at six months between the two arms of this study was found. In addition, the rate of neurological deaths in the SRS boost arm was within the 25–50% range reported in other surgery or SRS series [62]. Because of the known toxicity associated with WBRT and the lack of any difference in survival between SRS alone and SRS plus WBRT as described in previous sections, the use of SRS alone as initial treatment for patients with a limited number of lesions has been advocated by many. The difference in neurocognitive function between patients undergoing SRS alone and those undergoing SRS and WBRT has been investigated in two prospective randomized controlled trials [66,89]. In the study by Aoyama *et al.* [66], neurocognitive function was assessed by serial MMSE after SRS + WBRT or SRS alone. No statistical difference in MMSE scores was found between the two arms, nor was any statistically significant difference found in the rate of MMSE score deterioration after a median follow up of 5.3 months. However, the time to neurological deterioration was significantly longer in patients who received SRS + WBRT than in those who received SRS alone (16.5 months *versus* 7.6 months, *p* = 0.05). This was thought to reflect the higher number of intracranial recurrences in the SRS alone group (11 *versus* 3 patients, *p* < 0.0001). Five patients who underwent SRS + WBRT, but none in the SRS alone arm, suffered a radiation toxic event. Although not statistically significant, a trend of continuous neurocognitive deterioration became prominent after 24 months in long-term survivors after SRS and WBRT. These findings from Aoyama *et al.* corroborate those from Regine *et al.* and Li *et al.* in that WBRT may help to improve neurocognitive function in patients with brain metastasis through its therapeutic effects on a short-term basis [66,86,88]. Moreover, significant numbers of patients treated with SRS alone may experience recurrence with neurological symptoms, leading to the recommendation that WBRT be used whenever indicated [85]. However, the late toxicity in terms of neurocognitive function from WBRT in long-term survivors cannot be ignored and warrants further investigation, as such effects may be masked owing to the short survival time of many patients on these studies of mostly far less than two years. Recently, the effects of initial treatment with SRS alone or SRS combined with WBRT on learning and memory function were investigated in a prospective randomized study by Chang *et al.* [89]. Most patients in this study had NSCLC, 1-2 lesions, and RPA class I or II. The GPA indices between the two arms were also well balanced. This study was designed to detect a 5-point decline in the Hopkins Verbal Learning Test–Revised (HVLT-R). The study was stopped when a significant decline in the HVLT-R score at four months was observed in the SRS plus WBRT arm compared with the SRS alone arm after accrual of 58 patients. Overall, the total recall difference persisted at six months. The patients who received SRS + WBRT demonstrated greater declines in executive function as well. Increased intracranial failure was observed in the SRS alone arm, with approximately 87% of the patients requiring salvage therapy. However, the one year survival rate was higher in the SRS alone arm (63% *versus* 21%, *p* = 0.003), possibly because of earlier systemic therapy in the SRS group and greater systemic disease burden in the SRS + WBRT arm. The authors argued for the initial treatment to be SRS alone with close follow up since intracranial recurrences are likely to be asymptomatic if discovered in their early stages by imaging studies.

Given the evidence described above, WBRT does seem to have a toxic effect on neurocognitive function over time. However, neurocognitive deterioration can be observed only in long-term survivors. Therefore, WBRT may be omitted in patients with good functional performance status and limited numbers of metastases if those patients have limited extracranial disease and are aware of the risk of intracranial failure associated with SRS alone and the risk of potential neurological deficits as a result of such failures. Thus, we recommend offering SRS alone to patients who can be monitored closely (e.g., every two months) with MRI. SRS plus WBRT should still be given serious consideration for patients with good functional performance status with controlled extracranial disease and single brain metastasis given the observed survival benefit observed in RTOG 9508 [62]. In contrast, WBRT can actually improve neurocognitive function of patients with radiosensitive tumors, such as lung cancer, poor prognosis, and a short lifespan. Thus WBRT should be recommended for such patients. In recent years, donepezil, a drug used to treat Alzheimer’s disease, was shown to have a positive effect on the cognitive function of patients who underwent irradiation for brain tumors [90]. The potential role of memantine, an agent that blocks the pathologic stimulation of the *N*-methyl-d-aspartate (NMDA) receptor (a receptor involved in learning and memory), in alleviating neurocognitive deficits from WBRT is being investigated in a randomized Phase III study, RTOG 0614, with results pending [91].

Also worth mentioning is the potential contribution of anticonvulsants to the development of late neurological symptoms from WBRT [92]. Therefore, any systemic agents (e.g., anticonvulsants, steroids) that can possibly influence the symptomatic outcome from brain irradiation should be carefully assessed and controlled for in future prospective studies to reach firm conclusions regarding the incidence of late radiation toxicity from brain irradiation.

## 6. Prophylactic Brain Irradiation (PCI)

### 6.1. SCLC

SCLC is known for its high risk of early hematogeneous dissemination, especially to the brain. Upon initial diagnosis, up to 24% of patients may have brain metastases when MRI of the brain is included as part of the staging evaluation [93]. Most patients with SCLC will ultimately develop brain metastases if they live long enough. Prophylactic WBRT has been advocated by many to delay the development of brain metastases and reduce the rate of distant relapse in the brain [94,95]. Many older randomized studies have demonstrated statistically significant reductions in brain metastases from 16–73% to 0–13% with the use of PCI, although none was able to demonstrate any survival benefit [96,97,98,99,100,101]. This is mainly from the lack of patient stratification based on tumor stage (limited *versus* extensive) and response to definitive therapy. However, PCI was suggested to improve survival in patients who had a complete response (CR) to induction treatment in several retrospective studies [102,103,104]. Subsequent randomized studies of PCI have focused on patients who have achieved a CR after initial treatment. Although these studies could not demonstrate a survival advantage with PCI individually, a 5.3% increase in 3-year OS in patients who received PCI (*p* = 0.01) was detected when individual data from 987 CR patients enrolled between 1965 and 1995 into seven trials comparing PCI to observation were analyzed in a meta-analysis [105]. The Most of the patients were men (75%) with good performance status (97%) and limited-stage disease (86%). CR in the chest was assessed by chest X-ray, bronchoscopy, or thoracic CT. The cumulative incidence of brain metastasis at three years decreased from 58.6% in the observation group to 33.3% in the PCI group (*p* < 0.001). A trend toward decreased risk of brain metastasis was observed with increased radiation dose when four dose regimens were compared (8 Gy/1 fraction, 24-25 Gy/8-12 fractions, 30 Gy/10 fractions, and 36–40 Gy/18–20 fractions, *p* = 0.02). In addition, PCI seems to have a greater effect on the incidence of brain metastases if delivered sooner after induction therapy (*p* = 0.01). The association of PCI and a survival benefit was demonstrated again in CR patients in another meta-analysis of 12 randomized trials involving 1547 patients by Meert *et al.* [106]. Based on these meta-analyses, PCI became a part of the standard of care for SCLC patients in CR.

The survival advantage associated with PCI was also demonstrated in patients with extensive stage SCLC who had no response to four to six cycles of chemotherapy [107]. Disease-progression-free survival was significantly longer in the PCI group (14.7 weeks *versus* 12.0 weeks, *p* = 0.02), as was median survival (6.7 months *versus* 5.4 months, *p* = 0.003). The risk of symptomatic brain metastases was significantly decreased with PCI at one year (14.6% *versus* 40.4%, *p* < 0.001). Notably, brain imaging was not required for this trial. Therefore, many patients in the PCI group may have been treated for asymptomatic brain metastasis.

Based on the evidence summarized above, PCI should be offered to any patient with limited stage SCLC with CR after initial treatment, or extensive stage SCLC with any response after initial chemotherapy as the standard of care.

Although improvement was seen with PCI, a 33% incidence of brain metastases is still observed three years after PCI as demonstrated in a meta-analysis by Aupérin *et al.* [105]. Because of the poor prognosis associated with brain metastases after PCI and a possible dose-response effect observed in the same meta-analysis, a Phase III randomized prospective study was conducted by the PCI Collaborative Group to address the question of dose effects in patients with limited stage SCLC and CR after definitive therapy [108]. The standard dose of 25 Gy/10 fractions was compared to 36 Gy delivered in either 18 daily fractions or 24 twice-daily fractions in this study. No statistically significant difference in the total incidence of brain metastases was found at two years between the two dose groups (29% standard dose groups *versus* 23% higher dose group, *p* = 0.80). However, a significantly lower incidence of brain metastases as the first site of failure at two years was observed in the higher dose group (6% *versus* 12%, *p* = 0.005). The higher dose group had a lower two year overall survival rate (37% *versus* 42%, *p* = 0.05), which was most likely due to increased intrathoracic failure in this group relative to the standard dose group (48% *versus* 40% at two years, *p* = 0.02). These findings imply that intrathoracic disease control affects both the incidence of brain metastases after PCI and overall survival after multimodality treatment. On the other hand, these findings may also reflect the heterogeneous T and N categories of the patients among the study arms, possibly leading to the prevalence of poorer intrathoracic control for locally advanced tumors seen more commonly in one arm over another. Furthermore, the utility of higher doses for PCI is only rational in a select group of patients in whom intrathoracic disease is well controlled. Currently, 25 Gy delivered in 10 fractions is still recommended as the standard of care given the lack of evidence for increased intracranial control associated with higher doses and the concern over potential adverse effects on neurocognitive function from WBRT. However, other dose fractionation regimens are reasonable alternatives as well (e.g., 30 Gy in 15 fractions).

### 6.2. NSCLC

The development of brain metastases is also prevalent in NSCLC. In stage III patients, the incidence of brain metastases during the course of treatment can reach approximately ≥ 50% [109,110]. Nonsquamous histology, bulky mediastinal nodes (>2 cm), increased numbers of positive mediastinal nodes, involvement of several nodal stations, younger age, the use of neoadjuvant chemotherapy, and prolonged survival were all found to be associated with the increased incidence of brain metastases in various studies [110,111,112,113,114,115,116,117]. Brain metastasis is also usually the most common site of distant failure [118,119]. The results of selected studies on the incidence of brain metastasis after combined modality treatment are presented in Table 7.

**Table 7:** Incidence of brain metastases after combined modality treatments for non-small cell lung cancer (NSCLC).

	Stage	Treatment	Brain Metastases (%)		Median survival (mo)
			Overall Survival	Brain as the 1st site of failure	
Wang *et al.* [110]	III	S ± adjuvant chemo	38.1	26.5	29.5
Ceresoli *et al.* [114]	IIB/III	Chemo/RT ± S	29 (2 yr)	22	21
Robnett *et al.* [115]	II/III	Chemo/RT	30% (2 yr)	19	14.5
Komaki *et al.* [117]	II/III	RT	N/A	6-18	9
Mamon *et al.* [118]	IIIA	Chemo/RT/S	40 (3 yr)	34 (3 yr)	21
Germain *et al.* [119]	III	Chemo/RT	27	21	19.2
Carolan *et al.* [120]	III	Chemo/RT ± S	34.9	18.1	25.6

Abbreviations: mo: months; S: surgical resection; Chemo: chemotherapy; RT: radiotherapy; yr: years

**Table 8:** Prospective randomized studies evaluating PCI for non-small cell lung cancer.

	Primary Therapy	Brain		Overall		Neurocognitive Deficits Associated with PCI
		Failures (%)		Survival (%)		
		No PCI	PCI	No PCI	PCI	
Cox *et al.* [121]	RT	13	6	NA		Not formally assessed
Russell *et al.* [122]	RT	19	9	21	13	Not formally assessed
Umsawasdi *et al.* [123]	Chemo/RT ± S	27	4	~ 17.5% after 39 mo with or without PCI		Not formally assessed
Pöttgen *et al.* [124]	Chemo/ RT/S	34.7	7.8	16%-18% at 5 years with or without PCI		None after five years
Movsas *et al.* [125] (Outcome at one year)	RT/S ± Chemo	18	7.7	76.9	75.6	Immediate recall and delayed recall

Abbreviations*:* PCI: prophylactic cranial irradiation; RT: radiotherapy; Chemo: chemotherapy; S: surgical resection; mo: months

Because of the poor prognosis associated with brain metastases and its prevalence in locally advanced NSCLC, the potential role of PCI has been investigated in several studies (Table 8). Although found to significantly decrease the incidence of intracranial metastases by some [121,122,123,124,125], no randomized study has been able to demonstrate any survival benefit from PCI in locally advanced NSCLC. Thus, PCI is currently not a part of the standard of care for locally advanced NSCLC. However, the prognostic factors found in retrospective studies may guide the section of patients for future prospective randomized studies to identify a subgroup of patients for whom PCI can lead to a survival benefit.

### 6.3. Neurocognitive Functioning after PCI

Significant late toxicity has been reported for patients with SCLC treated with PCI and concurrent chemotherapy or treated with large fractions [126]. Two randomized controlled trials have specifically examined neurocognitive function after PCI for SCLC [127,128]. Arriagada *et al.* randomized 300 patients with SCLC in complete remission to PCI *versus* observation in a prospective study from France [127]. Neuropsychological assessment was performed at baseline and on follow up to 48 months by a neurologist for 229 patients. The results of 83% baseline testing were considered normal in both arms. Overall, no difference was found between the two treatment arms in terms of higher functions, mood, walking, cerebellar function, tendon reflexes, sensibility, or cranial nerve function after two years. No statistically significant difference in the two year rates of abnormalities between the two arms was observed. In a similar study by Gregor *et al.* [128], 314 patients with limited stage SCLC in CR were randomized to PCI or no PCI. Neurocognitive function was formally assessed with a battery of tests, including the national adult reading test, the paced auditory serial addition task, the Rey Osterrieth complex figure test, and the auditory verbal learning test. The quality of life and anxiety and depression were assessed with Rotterdam symptom checklist and the Hospital anxiety and depression scale. Tests of cognitive function revealed cognitive impairment in 24% to 41% of patients in each group. However, no significant difference was found between the two arms at the baseline. Furthermore, no difference between the two arms was observed in neurocognitive function or gross quality of life, level of anxiety, or depression at one year. Findings were similar in a recent prospective randomized trial evaluating PCI in locally advanced NSCLC by Pöttgen *et al.* [124]. Of 11 evaluable long-term surviving patients, no deficits in attention, memory, associative learning, and information processing were found by using a battery of neurocognitive tests in patients who received PCI and those who did not. However, this lack of difference can also be explained by the small number of patients. In contrast, increased decline in both immediate and late recall was observed in the Hopkins verbal learning test at one year when patients with stage III NSCLC underwent PCI 30 Gy in 15 fractions in the Phase III prospective randomized study RTOG 0214 [125]. These findings may clarify neurocognitive function after PCI as the findings mature. The inclusion of neuropsychometric testing has not been common practice in the past. Its inclusion in current and future trials will enhance our understanding of the long-term neurocognitive effects of PCI and WBRT in general.

## 7. Local Therapy for Synchronous, Solitary Brain Metastasis from NSCLC

Some proportion of patients with NSCLC present with synchronous brain metastasis. Five year overall survival rates of over 20% have been reported after both the brain and the primary site were treated aggressively (Table 9).

**Table 9:** Survival outcome from definitive therapy in patients with solitary brain metastasis.

	n	Stage of Chest Disease	Brain Therapy	Definitive Therapy	5 Year Overall Survival (%)
Hu *et al.* [129]	84	I-III	S/SRS	RT ± Chemo	7.6
					Stage I: 50 (3 year)
Flannery [130]	42	I-III	SRS	S ± chemo/RT	21
Bonnette *et al.* [131]	99	I-III	S	S	11
Billing *et al.* [132]	28	I-III	S/WBRT	S ± chemo/RT	21.4
Ampil *et al.* [133]	72	I-III	WBRT/SRS	RT	13% (1 year)
Lo *et al.* [134]	18	I-III	S/SRS	S	27

Abbreviations: S: Surgical resection; SRS: stereotactic radiosurgery; Chemo: chemotherapy; WBRT: whole brain radiotherapy

After SRS, overall survival was significantly higher for those given definitive (as opposed to non-definitive) thoracic therapy in a study of 42 patients by Flannery *et al.* [130]. Furthermore, higher survival was demonstrated to be associated with early stage disease in the chest and better KPS [129,130,132]. A survival benefit was also observed for patients with more than one synchronous brain metastasis when those patients also had good functional performance status and received thoracic therapy [135,136,137]. All of these findings support the delivery of local therapy to the chest for patients with good functional performance status and limited numbers of brain metastasis. However, the survival benefit from local therapy still needs to be validated in a prospective randomized controlled trial.

## 8. Future Investigations

Given the lack of any survival benefit demonstrated for adjuvant WBRT after surgical resection or SRS and its potential neurotoxicity, the utility of SRS to provide a boost dose to the tumor bed after craniotomy for patients with limited numbers of brain metastases has been investigated in recent years. In a study of 72 patients (43% NSCLC) with 1-4 brain metastases and 76 cavities after surgical resection, a median dose of 18.0 Gy was delivered to the median 79% isodose line at the periphery of the tumor bed [138]. The actuarial local control rate in this study was 79% at two years, and the distant control rate was 47% at 12 months. Three patients underwent surgical resection of a region of necrosis. Use of less conformal plans translated into a local control rate of 100%, thus the authors recommended a planning target volume margin of 2 mm around the resection cavity. In a similar study of 52 patients (46% NSCLC) with up to four lesions, a local failure rate of 7.7% was observed after a median follow up of 13 months [139]. The distant failure rate was 44% after a median of 16 months after resection, and the median survival was 15 months. Similar results have been reported in other single-institution studies [141,142]. Equivalent local control between surgery followed by adjuvant WBRT or SRS is suggested by these small studies, but these findings remain to be validated in a prospective randomized study. However, the risk of distant recurrence remains high with adjuvant SRS alone, making this approach inappropriate for patients with solitary brain metastasis, good functional performance status, and primary disease controlled locally because of the potential for aggressive treatments to improve survival in these patients, especially for patients with lung cancer [142,143]. Therefore, WBRT may still be warranted in patients with good prognosis in addition to surgery and adjuvant SRS, if the potential toxicity is tolerable. The feasibility of this approach was investigated in a small study of 27 patients (70% NSCLC); the actuarial two-year local control was 94%, and the two year actuarial incidence of new brain metastasis was 30% [144]. Only one patient required reoperation for symptomatic radiation necrosis at 16 months after treatment. The median survival was 17.6 months. Whether this approach will lead to a survival benefit still requires further investigation in a randomized trial.

As mentioned previously, SRS can spare adjacent normal tissue by achieving a sharp dose gradient at the periphery of the tumor target volume. However, this advantage is diminished with large lesions. To spare normal brain tissue, the dose delivered needs to be decreased to avoid potential neurotoxicity [51,145]. The tumor response is usually impaired as a result of this [146]. Therefore, fractionated stereotactic radiotherapy has been proposed owing to the advantages of reoxygenation of hypoxic cells within large lesions and significant increases in late-responding tissue sparing if the radiation dose is fractionated [147]. Thus, the therapeutic ratio can be significantly increased when large brain metastases are treated with a high dose delivered in several fractions with a stereotactic set-up. This concept has been validated in a small study, in which patients with large brain metastases (average volume 21.2 cm^3^) were treated safely with fractionated stereotactic radiotherapy, and a local control rate of 83% has been achieved [148]. These findings were confirmed in a larger retrospective study of patients with large brain metastases treated with this technique [149]. Similarly, many single-institution studies have shown excellent clinical outcome and toxicity profile from fractionated stereotactic radiotherapy with or without use of a frame (Table 10). These studies suggest that local control seems to be related to tumor size [155] and that intracranial control outside of the treated area seems to be poor when fractionated stereotactic radiotherapy is used alone. However, its combination with WBRT was shown to be feasible with good intracranial control and toxicity profile [156,157], which may be a better option for patients who present with neurological deficits from large, but few, brain metastases. However, the patient selection criteria for this technique, alone or as a boost, needs further investigation in prospective studies. Also, it is important to be aware that a bigger margin than that used for SRS may be needed when patients are not precisely immobilized.

**Table 10:** Selected studies of fractionated stereotactic radiotherapy.

	N (patients/ metastases)	Dose (Gy)	Response	1-year Overall Survival (%)	Distant Failure in the Brain	Toxicity
De Salles *et al.* [148]	26/41	6 Gy × 2-3	LC 83%	NR	NR	NR
			FU 2-18 mo			
Nishizaki *et al.* [149]	71/148	7.8-30.1 Gy/1-3 fractions	Median FU: 11 mo	47	35.2 % at a median FU of 6.6 mo	No permanent symptoms from radionecrosis
			LC: 83%			
Manning *et al.* [150]	32/57	6-12 Gy × 3;	31% ≤ 25%	44	13% after ≥ 6 mo	Seizure: 12%
		WBRT for all patients	31% > 25%			Radionecrosis: 6%
			16% PD			
Aoyama *et al.* [151]	87/159	8.75 Gy × 4	1 yr LC: 81%	39	60% at 1 yr	Symptomatic radionecrosis: 2.7%
			2 yr LC: 69%			
Aoki *et al.* [152]	44/65	18-30 Gy/ 3-5 fractions	1 yr LC: 71.9%	50.8	31% at 1 yr	No severe complications
Ernst-Stecken *et al.* [153]	51/72	6 Gy × 5	1 yr LC: 76%	Median survival: 11 mo	NR	Increased radionecrosis if V_4Gy_ > 23 cm^3^ (70% *vs.* 14%, *p* = 0.001)
		7 Gy × 5				
		SRT alone or with WBRT				
Fahrig *et al.* [154]	150/228	6-7 Gy × 5	CR: 46%	66	NR	10% toxicity; 1 symptomatic hemorrhage from melanoma; 2 had to be operated on for radionecrosis;
		4 Gy × 10	31%			
		5 Gy × 7	47%			
			Median FU: 28 mo			No grade 5 toxicity;
						No toxicity with 4 Gy × 10.
Kwon *et al.* [155]	36/66	20-36 Gy/ 4-6	1 yr LC: 68.2%;	43.9	25.9% at a median FU of 6.47 mo	Radionecrosis: 5.8%
			Tumors < 1 cm^3^ had better LC			
Lindvall *et al.* [156]	61/77	8 Gy × 5	LC: 84% (no WBRT); 100% (WBRT); mean 3.7 mo after SRT	Mean survival from time of SRT: 6.1 mo	25% after a median of 3.7 mo after SRT alone, but none in WBRT + SRT patients who were followed radiologically	4.7% radionecrosis after SRT + WBRT
		± WBRT				
Giubilei *et al.* [157]	30/44	6 Gy × 3	1 yr LC: 86.1%	36.6	12.1% at 1 yr	No acute or late complications
		8 Gy × 4				
		WBRT for all patients				

Abbreviations: CR: complete response; FU: follow up; NR: not reported; LC: local control; mo: months; WBRT: whole brain radiotherapy; PD: progressive disease; SRT: stereotactic radiotherapy; yr: year

In patients with good prognostic factors, such as 1–3 brain metastases, RPA class 1–2, controlled extracranial disease, GPA of ≥ 2.5, young age, and high KPS scores, aggressive treatment of brain metastasis with WBRT followed by a regular external beam boost to the gross tumor or the surgical bed has been investigated. This approach has been consistently shown to be associated with increased local control comparable to that reported for WBRT + SRS, as well as a median survival time of over 12 months, significantly better than WBRT alone with or without surgical resection [158,159]. Radiation delivered as a simultaneous integrated boost with intensity-modulated radiotherapy has been shown to produce a sharper dose gradient than that from WBRT followed by SRS for the treatment of brain metastasis [160,161]. This leads to improved normal tissue sparing owing to the ability to optimize the dose to the normal brain and to account for dose spillage from the boost dose to the adjacent brain tissue in the planning of WBRT. This not only spares more normal brain tissue but also shortens treatment time for patients with multiple lesions. The feasibility of this approach has been demonstrated in a single-institution Phase I study through the use of helical tomotherapy, which combines delivery of intensity-modulated radiotherapy with megavoltage CT imaging for precise radiation delivery through image guidance [162]. In this study, 48 patients (50% of whom had lung cancer) were treated with WBRT 30 Gy in 10 fractions and a simultaneous integrated boost to the brain metastases safely escalated from 5 to 30 Gy in 10 fractions. No grade 3–5 dose limiting toxicity was encountered. However, this study had a median follow up of only 7.72 months and a median overall survival time of only 5.29 months. Given the small number of patients in this study, no firm conclusions can be made regarding tumor response and survival outcome. Worth mentioning is the application of helical tomotherapy in the treatment of recurrent brain metastases from lung and breast cancer with a simultaneous integrated boost. The safe delivery of 30 Gy to the gross disease and 15 Gy to the whole brain in 10 fractions for up to 11 lesions was reported by Sterzing *et al.* [163]. In this report, an excellent dose conformality index was achieved, and no severe toxicity was observed; the patients remained recurrence-free at six and 12 months of follow up. Based on this limited evidence, the simultaneous integrated boost approach may be an excellent treatment approach with intensity modulation of doses to the target and the adjacent brain tissue. Depending on the degree of immobilization, no standard has been established regarding the planning target volume margin for gross disease, and margins from 0 to 10 mm have been reported [158,161,162,163]. Further conclusions regarding this matter can be made as the existing data mature. Recently, excellent dose sparing of radiosensitive structures, such as the hippocampus, was reported when brain metastases were treated with the simultaneous integrated boost approach, which further supports the use of this approach for normal tissue sparing [164,165].

Other future work involves targeting therapy to the epidermal growth factor receptor (EGFR) family of four homologous receptors, EGFR (ERBB1), HER-2/neu (ERBB2), HER-3 (ERBB3), and HER-4 (ERBB4). EGFR activation leads to receptor tyrosine-kinase activation and activation of a series of downstream signaling activities that mediate tumor cell proliferation, migration, invasion, and the suppression of apoptosis [166]. As a result, inhibiting EGFR by binding its intracellular adenosine triphosphate-binding site with small-molecule tyrosine kinase inhibitors has been investigated as a treatment strategy for NSCLC [167]. Among these inhibitors, erlotinib has shown a survival benefit when combined with chemotherapy for advanced stage NSCLC [168]. However, tumor response is mainly limited to patients who possess somatic mutations in the kinase domain of the EGFR gene [169]. These patients are usually of East Asian descent, female, nonsmokers, with adenocarcinoma [170]. In a small series of 41 patients with brain metastasis from lung adenocarcinoma, gefitinib was shown to have antitumor activity (10% major response). Intracranial control was associated with previous WBRT [171]. In another study by Kim *et al.* [172], median progression-free survival and overall survival times of 7.1 and 18.8 months were observed in East Asian, nonsmoking patients with lung adenocarcinoma and asymptomatic synchronous brain metastasis after treatment with either gefitinib 250 mg or erlotinib 150 mg once daily. Both studies suggest a potential role for EGFR inhibitors in the treatment of brain metastases. Additive effects may be produced when both EGFR inhibitors and WBRT were delivered, as patients who received both treatments have better disease control and longer overall survival [171,172]. Several studies have demonstrated increased response to EGFR inhibitors, as well as prolonged time to intracranial progression and improved overall survival, in patients with mutations in the EGFR gene [173,174]. The presence of EGFR mutations was shown to enhance radiation response; for patients with EGFR mutation and brain metastases from lung adenocarcinoma, WBRT delivered concurrently with EGFR inhibitors produced a response rate of 84% [175]. However, severe toxicities, including grade 5 interstitial lung disease, have been reported in patients treated with concurrent erlotinib and WBRT [176,177]. The unexpected lung toxicity from EGFR inhibitors needs further investigation for the safe administration of these drugs.

## 9. Conclusions

**Figure 1:**
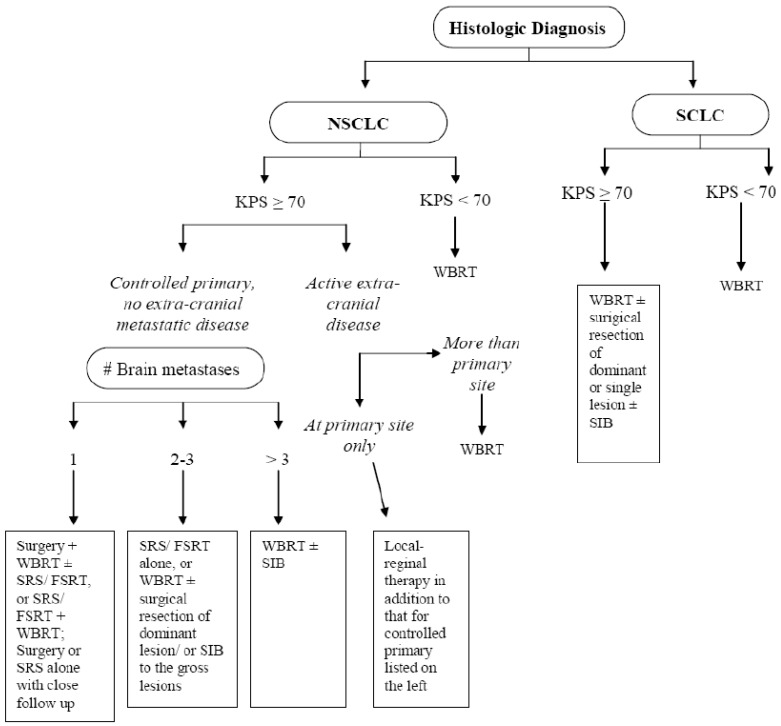
Algorithm for the initial treatment of brain metastases from lung cancer.

In summary, patients with brain metastases from lung cancer have several treatment options, which are summarized in Figure 1. The choice of treatment will greatly influence the overall prognosis for patients with advanced stage lung cancer. Based on current evidence, combined modality treatment of brain metastases has greatly improved the survival of patients with single lesions, good functional performance status, and controlled extracranial disease, as demonstrated in prospective randomized studies. Neurocognitive deterioration remains a concern for patients with excellent functional performance status who are receiving WBRT ± SRS. However, radiotherapy may improve neurocognitive function in a select group of patients who present with neurological impairment from brain lesions at baseline shortly after treatment. PCI for SCLC is currently part of the standard of care, but PCI for NSCLC is still investigational. Local therapy should be considered for patients with early stage intrathoracic disease and brain as the sole site of metastasis. To further improve treatment outcome for brain metastasis, options including an SRS boost to the surgical bed alone, fractionated stereotactic radiotherapy, and WBRT with a simultaneous integrated boost are currently under investigation. Among these options, fractionated stereotactic radiotherapy allows the delivery of a high dose in a few fractions, which is a more biologically sound approach for large lesions. This technique can also potentially decrease the toxicity from SRS because a lower dose is delivered per fraction over multiple fractions, thus greatly reducing the risk of late normal tissue damage. WBRT with a simultaneous integrated boost allows dose optimization such that a high dose is given to the target volume while the dose delivered to the whole brain is kept below a certain threshold. This achieves increased tumor dose, while sparing as much normal brain tissue as possible to prevent neurological toxicity from radiotherapy. Such an approach holds great promise in the future. Radiosensitization is not currently indicated clinically. However, the use of EGFR inhibitors ± WBRT has demonstrated good response of intracranial disease in patients with EGFR mutations, and this strategy also warrants further clinical investigation.
